# Developing and validating a measure of L2-specific emotion regulation strategies

**DOI:** 10.1371/journal.pone.0345751

**Published:** 2026-04-08

**Authors:** Huiyuan Gu

**Affiliations:** School of Languages and Humanities, Guangzhou College of Technology and Business, Guangzhou city, China; Wenzhou Medical University, CHINA

## Abstract

**Background:**

Despite recent scholarly interest in the regulation of academic emotions in second language (L2) learning, L2 studies have primarily relied on domain-general instruments to measure L2 emotion regulation (ER) strategies. In response, this study created and validated a domain-specific instrument, the L2 Emotion Regulation Strategies Questionnaire (L2ERSQ).

**Methods:**

Two waves of data were collected from 811 Chinese tertiary EFL learners. The factorial structure, reliability, and validity of the L2ERSQ were assessed through a series of analyses such as exploratory factor analysis and confirmatory factor analysis.

**Results:**

Exploratory factor analysis and confirmatory factor analysis have confirmed a 7-factor structure of the questionnaire, which possessed satisfactory construct reliability and validity. ER strategies significantly correlated with L2 enjoyment and boredom within both waves of data, demonstrating the concurrent criterion validity of the L2ERSQ. Model comparisons indicate that the L2ERSQ possesses longitudinal measurement invariance, showing its potential for longitudinal studies of emotion regulation.

**Conclusion:**

This study demonstrates the reliability and validity of a questionnaire assessing L2-specific emotion regulation strategies. The newly developed instrument enriches the existing taxonomy of emotion regulation strategies by distinguishing between value upgrading and value downgrading within the broader category of value appraisal. The findings indicate that some emotion regulation strategies (e.g., value upgrading) are adaptive, while others (e.g., value downgrading) are ambivalent. Implications for research on L2 emotion regulation and the development of interventions to enhance emotion regulation are discussed.

## Introduction

Learners may experience various emotions in their second language (L2) learning [1]. Such emotions could exert complicated impacts on L2 learners’ academic engagement [2–5], motivation [6–8], self-regulated learning [9], and academic achievement [10]. Positive emotions generally broaden cognitive and attentional resources [11], whereas the impacts of negative emotions are more complicated [12]. For example, while anxiety often disrupts academic performance, it can also serve as a motivator for learners if the fear of future failures is transformed into a greater commitment to learning [13–15]. In addition to academic engagement and performance, the well-being and happiness of L2 learners also depend on how they emotionally respond to L2 learning activities or outcomes [16–18]. Emotion regulation is the key to adapting emotions to suit personal goals and benefits. Despite the large body of investigations in emotion generation in L2 learning, relatively few studies have considered how emotions can be regulated and which strategies are adaptive or maladaptive for managing these emotions [19]. In contrast to the numerous L2-specific emotion questionnaires, validated L2-specific instruments have been surprisingly scarce. The lack of reliable questionnaires has limited scholarly research on emotion regulation in L2 learning, particularly in light of the ongoing validation crisis in measurement instruments [20]. While L2 researchers can adapt existing questionnaires of ER strategies originally developed for clinical purposes [21] or domain-general academic context [22], such instruments must be modified to align with the respective study context by changing item content or removing items [7,15,23]. Questionnaire modification costs extra time and resources, complicates the procedures of research, and poses a threat to the reliability and validity of the measures [24]. Modified measures of ER strategies whose psychometric properties are not validated also weaken confidence in the findings about emotion regulation. As a timely response to recent calls for research on emotion regulation [19,25], this study developed a domain-specific questionnaire of emotion regulation strategies in L2 learning, the L2 Emotion Regulation Strategies Questionnaire (L2ERSQ). The validation results not only established the newly created questionnaire but also provided extra evidence on its longitudinal measurement invariance. This study is guided by three questions:

RQ1. What is the underlying factorial structure of L2ERSQ?
RQ2. To what extent is L2ERSQ psychometrically reliable and valid?
RQ3. Does L2ERSQ possess longitudinal measurement invariance?

## Literature review

### Emotions in L2 learning and their regulation

Emotions in language learning have garnered sustained research interest [8,18,25–31]. L2 researchers have extensively explored the nuanced influence of emotions on the behaviors and outcomes of L2 learners [9,32]. These studies not only revealed how positive emotions can broaden the cognitive sources for L2 learning but also framed negative emotions (e.g., anxiety) from a positive psychology perspective and recognized their potential as a productive learning experience [13,33]. Other investigations have considered the influence of language learning on the well-being of L2 learners [18,34,35]. It has been argued that bilinguals have an emotional advantage over monolinguals [36], and that multilinguals tend to experience less communicative anxiety [37]. Emotional well-being or happiness has been increasingly recognized as a desirable outcome of language learning [17,18]. The critical question is how to promote emotional well-being. One strand of research aims to identify the external factors that affect L2 emotions [38,39], and the other takes an individual differences perspective to explore how learner-internal factors may shape L2 emotions [1]. One primary learner-internal factor is how L2 learners exercise emotion regulation strategies.

Emotion regulation refers to the processes by which individuals influence which emotions they have, when they have them, and how they experience and express these emotions [40]. As emotion regulation has become a key topic in L2 research, the strategies that are proper for emotion regulation have also attracted growing attention. The emerging body of relevant research has considered two critical issues. The first is what strategies L2 learners employ to regulate their emotions [41], and the second is which strategies are effective [23]. For example, L2 researchers have explored the types of ER strategies using qualitative methods such as interviews and observations [6–8]. The previous studies have revealed a wide range of ER strategies employed in the L2 context. By contrast, quantitative studies on the effectiveness of emotion regulation have typically relied on a relatively narrow taxonomy of emotion regulation strategies adapted from existing questionnaires. For instance, Namaziandost and Rezai [7] adapted the Academic Emotion Regulation Questionnaire (AERQ). Their study demonstrated that emotion regulation can enhance L2 motivation, learner autonomy, and the L2 learning experience. Alqarni [42] adapted the Emotion Regulation Questionnaire (ERQ) developed by Gross and John [40] to explore the effectiveness of ER strategies. His study showed that reappraisal was positively linked with higher levels of enjoyment and stress, whereas suppression was found to enhance enjoyment and alleviate stress. Additionally, his study revealed that reappraisal indirectly predicted English proficiency through enjoyment and stress, while suppression indirectly predicted English proficiency only through stress. Man et al. [15] explored the effectiveness of five families of ER strategies by adapting items from the Academic Emotion Regulation Questionnaire (AERQ) and the Emotion Regulation Questionnaire (ERQ). They found that situation modification and response modulation are adaptive ER strategies, whereas situation selection and cognitive change are maladaptive ER strategies. Attention redirection was found to be positively related to both positive and negative emotions. While these studies provide valuable insights into the effects of emotion regulation strategies, their results are inconsistent. Notably, the role of cognitive reappraisal, or cognitive change, particularly merits attention. Emphasizing or downplaying the importance of language learning can lead to different emotional responses, such as increased or decreased anxiety. There is a need to differentiate between these two types of cognitive reappraisal. In addition, the instruments employed to measure ER strategies were all adapted from questionnaires developed for contexts such as clinical or general academic settings. Because emotions and their generation are domain-specific, the domain-general measures of ER strategies should be carefully evaluated before they can be used in the L2 context. The previous studies [e.g., 42] have all reported changing or removing items from their adapted questionnaires. However, the psychometric properties of the adapted measures have not been assessed with an independent sample. If a reliable instrument is available, relevant studies can save the trouble of adjusting or validating the measures of ER strategies in L2 learning. More importantly, an L2-specific instrument that possesses longitudinal invariance can respond to the repeated calls for longitudinal research designs.

### Measures of emotion regulation strategies

Emotion regulation encompasses both intrinsic and extrinsic mechanisms that enable individuals to identify, observe, assess, and modify their emotional responses [22]. According to the Process Model of Emotion Regulation [43], emotion regulation can be automatic or controlled and can address both positive and negative emotions. It can influence the latency, rise time, intensity, and duration of emotional responses, as well as behavioral and physiological reactions, depending on individual goals [40]. The existing literature has provided three questionnaires of ER strategies that have been widely adapted to L2 research.

The first is the CERQ created by Garnefski et al. [21]. The CERQ exclusively encompasses cognitive strategies, as opposed to behavioral strategies, employed by individuals exhibiting symptoms of depression and anxiety in reaction to threatening or stressful situations. The cognitive strategies in CERQ were primarily oriented toward coping with negative, intense emotions. Based on a sample of adults with emotional problems, Garnefski and Kraaij [44] discovered that individuals employed adaptive strategies, such as cognitive reappraisal, more often than they employed less adaptive strategies, such as self-blame. The CERQ has recently been adapted to examine ER strategies in L2 learning [45].

The second is the ERQ, a 10-item questionnaire of two emotion regulation strategies (i.e., cognitive reappraisal and expressive suppression) [40]. Experimental and correlational research consistently indicates that reappraisal serves as an adaptive strategy that enhances well-being and diminishes negative emotions, while suppression is deemed maladaptive in the regulation of negative emotions [46]. This questionnaire has started to inform L2 research [47], although the findings partially differ from prior research. For example, Alqarni [42] found that the use of reappraisal positively predicted stress, while suppression positively predicted enjoyment. The differing results may be attributed to the different contexts of the studies. ERQ was developed to investigate short-term emotion regulation in general contexts. In L2 learning, reappraising the objects that invoke emotions can have either a positive or a negative affective outcome [1,28]. For example, emphasizing the importance of a task could simultaneously raise anxiety and reduce boredom. Unfortunately, the ERQ was ambiguous about value reappraisal. The stem for the reappraisal scale is, “I change the way I think about …”. When it comes to L2 learning, learners might change their way by upgrading or downplaying the importance of an activity or its outcome. This ambiguity in the reappraisal scale might solicit different responses from L2 learners. There is a need for a more refined approach to reappraisal.

The third measure is the AERQ, a domain-general questionnaire created by Burić et al. [22]. In contrast with ERQ, AERQ measures a more comprehensive set of ER strategies, covering the various stages of emotion regulation. These include avoiding certain situations, developing competencies, seeking social support, redirecting attention, reappraisal, suppression, respiration, and venting. Burić et al. [22] found that, out of eight strategies, only four positively correlated with enjoyment and one negatively correlated with boredom; seven strategies were positively correlated with anxiety. This questionnaire has also been adopted as a major instrument in recent L2 research but with mixed results. For example, Namaziandost and Rezai [7] showed that all eight strategies positively predicted L2 motivation and learning experiences, whereas Wang et al. [23] found that avoiding, venting, and suppression were positively related to anxiety. It is worth noting that at least six sub-scales of the AERQ were oriented toward negative emotions. This orientation, more aligned with coping strategies in response to negative events, does not fully reflect learners’ proactive role in building resources and skills that would allow them to reap the best of future positive events.

In summary, none of the questionnaires currently employed in L2 research have been developed to measure ER strategies specific to L2 learning. The existing evidence produced by recent studies [e.g., 42], although limited, suggests that the use of ER strategies in L2 learning could vary from its use in other, more general contexts. For example, anxiety related to language learning is not always detrimental to language learning outcomes or learners’ emotional well-being. Studies in second language acquisition have shown that anxiety, when maintained at a reasonable level and properly regulated, can positively influence language achievement [13,14,33]. Emotional well-being does not preclude the presence of anxiety [18]. Emotional well-being is a balance of positive and negative emotions [48]. Emotion regulation can help manage the level of anxiety and contribute to this balance of emotions. In L2 learning, learners do not need to completely eliminate anxiety from their learning process. Instead, to enhance performance, they may intentionally maintain a moderate level of anxiety to promote greater learning engagement. More importantly, the positive psychology in L2 learning has increasingly emphasized the value of proactive, adaptive efforts to foster positive emotions [49]. The emphasis on positive emotions and emotional well-being differs from clinical psychology or prior L2 studies, which focuses on individuals’ strategies to cope with distress or intense negative emotions. In this sense, we need an L2-specific questionnaire that covers the strategies that can be employed across the stages of emotion regulation, fully considers the fostering of positive emotions in L2 learning, and possesses longitudinal measurement invariance. As a timely response to such a need, this article reports on designing and validating a domain-specific questionnaire, the L2 Emotion Regulation Strategies Questionnaire (L2ERSQ).

### Theoretical framework

This study was informed by an integrated model of emotion regulation in achievement situations (ERAS) proposed by Harley et al. [50]. This model was developed by integrating the propositions from the control-value theory (CVT) [12], an achievement emotion-generation theory, and the process model of emotion regulation (PMER), an emotion-regulation theory. The ERAS model delineates a four-phase framework (situation, attention, appraisal, response) for the elicitation of achievement-related emotions. The process commences with an achievement context that captures attention, subsequently prompting evaluations concerning its influence on individual objectives. The model identifies five families of ER strategies: (a) *situation selection* represents a deliberate choice of a situation that elicits desirable emotions and avoidance of a situation that invokes undesirable emotions; (b) *situation modification* pertains to the alteration of a given context through the adjustment of tasks or learning environments, as well as the enhancement of self-competence, so as to facilitate the experience of positive emotions and mitigate negative emotional responses; (c) *attentional deployment* refers to the process of directing one’s attention as a means of regulating emotional responses; (d) *cognitive change* adjusts one’s appraisals of control and value to modify emotional impact; (e) *response modulation* aims to change the experience or expression of emotions after they arise. According to the ERAS model, the desirability of an emotion is based on its fit with the situation, not just its pleasantness. This is because individuals may regulate their emotions for hedonic reasons (seeking to reduce unpleasant states and enhance pleasant ones) or for instrumental reasons tied to academic success [50]. Negative emotions, including anxiety, have the potential to improve L2 performance when effectively managed [33]. The ERAS model also includes propositions about specific ER strategies regarding their flexibility and effectiveness. ER strategies are most flexible in individual learning outside the classroom and most constrained in test-taking contexts. It has been posited that situation selection and situation modification, attention deployment, and cognitive change can upregulate positive emotions and downregulate negative emotions. Reappraising control may be more effective than downgrading task values in reducing anxiety because downgrading the value can simultaneously reduce motivation. Response modulation is the least instrumental in relieving negative emotions such as anxiety and boredom but most effective in lowering expressions of enjoyment. These propositions about ER strategies have not been systematically examined [51], possibly because of the lack of psychometrically reliable and valid instruments to measure ER strategies, especially in the L2 context. This study tests these propositions in an L2 context.

## Method

The present study adopted a quantitative instrument development design grounded in a postpositivist paradigm. Postpositivism assumes that while reality exists independently of human perception, it can only be approximated through scientific methods and empirical investigation. Guided by this stance, the study employed a systematic, multi-stage process to develop and validate an L2-specific questionnaire of emotion regulation strategies. The process included theory- and literature-based item generation, exploratory and confirmatory factor analyses to establish construct validity, correlation analysis to assess criterion-related validity, and measurement invariance testing to examine the instrument’s stability across time.

### Participants and the context

The participants in this research were selected utilizing purposive and convenience sampling techniques. Academic emotion regulation typically occurs in response to challenging or significant achievement-related activities or outcomes [50]. Consequently, this study focused on a test-preparation context in which students attended English courses prior to undertaking a critical English test. Utilizing the authors’ social networks, a cohort of 811 second-year students (222 males, 589 females) from a university in northern China was approached for this investigation. They were, on average, 19.6 years old (*SD* = 0.79). All the participants learned English as a foreign language for more than six years. Their English proficiency was considered intermediate based on English placement tests.

The study was approved by the Research Ethics Committee of Guangdong University of Foreign Studies (Approval No. GWGF-REC-202502). Teachers of the participants helped distribute the questionnaires to WeChat groups. The participants were informed of the research project. The questionnaire included a statement whether the participants were clear about their rights and the option to withdraw from the study. They gave their informed consent in the electronic questionnaire before completing it. The recruiting started from March 6^th^ 2025 to June 27^th^ 2025.

These students were preparing for a national standardized English proficiency test, the College English Test Band 4 (CET4). Successfully passing this test is regarded as indicative of an intermediate level of English proficiency. The CET4 certificate serves as a validation of English proficiency for non-English majors seeking employment in China. While obtaining the CET4 certificate is not a compulsory requirement for graduation, the university encourages students to pass the CET4 exam. Generally, students prepare for the CET4 using two methods. The first method involves attending English classes, where they are taught language skills such as reading and writing. While the curricular activities are not exam-oriented, the language skills taught in English classes are highly relevant to the CET4 test. The final exam for the English courses is similar to CET4, except that the listening test is often not included. Corrective feedback is one of the primary forms of teacher support in English classes. The second method involves practicing test-taking skills by working on past exam papers and sample test materials. Some students prepare for tests by working in pairs or groups.

### Instrument

The design of the L2-specific questionnaire of ER strategies (L2ERSQ) was inspired by the ERAS model [50]. The items for L2ERSQ came from three sources: (a) items adapted from Burić et al. [22], (b) self-developed items inspired by the relevant literature, and (c) items proposed by a panel of experts in survey studies in applied linguistics and by respondents in the pilot study. The items were written in Chinese. The created questionnaire initially included forty-three items. Three items were removed because the expert panel complained about item overlap and redundancy. One item was rephrased as suggested by respondents participating in the pilot study. The revised scale of ER strategies contained forty items (see Appendix A in S1 Appendices). To assess the criterion validity of the L2ERSQ, the participants’ L2 anxiety, enjoyment, and boredom were measured using four items for each emotion (see Appendix B in S1 Appendices). The items were adapted from the L2 classroom emotion sub-scales developed by Shao et al. [1]. In this study, the measures of the three emotions possessed good reliability at the two data collection time points: Anxiety (α = 0.918 and 0.917, respectively), enjoyment (α = 0.897 and 0.895, respectively), and boredom (α = 0.865 and 0.879, respectively).

### Data collection and analysis

The questionnaire was distributed to the participants through an online platform named *Wenjuanxing* (https://www.wjx.cn/). The participants were informed of the research purpose, and informed consent was solicited. The questionnaire was completed at two separate time points, with a duration of six weeks in between. Data curation was conducted prior to formal data analysis. After removing incomplete and straight-lining responses, the first wave of data included 695 participants (159 males and 536 females), and the second wave included 645 participants (162 males and 483 females). A total of 552 students (130 males and 422 females) participated in the survey for both time points.

The data analysis was performed in four systematic phases. The initial phase involved deciding on the factorial structure of the questionnaire through principal component analysis with varimax rotation. The first wave of data was utilized for this purpose. The second phase entailed performing a confirmatory factor analysis (CFA) with the second wave of data to validate the construct validity of the identified factorial structure. In this phase, factor loadings, Cronbach’s alpha, composite reliability (CR), and average variance extracted (AVE) were computed to assess the reliability and validity of the scale. Additionally, a series of conventional fit indices were employed to evaluate the overall model fit: the comparative fit index (CFI), and the Tucker-Lewis index (TLI), the root mean square error of approximation (RMSEA), and the standardized root mean square residual (SRMR). CFI and TLI values greater than 0.90 and RMSEA and SRMR values less than 0.08 indicate acceptable model fit [52]. In the third step, both concurrent and predictive criterion validity were assessed. The former considers how well ER strategies correlate with L2 emotions to be regulated at the same point of measurement, and the latter reflects how well ER strategies predict L2 emotions at a later measurement occasion. The two waves of data were used to assess concurrent validity by calculating the correlations between ER strategies and L2 emotions at each time. Finally, this longitudinal dataset was employed to validate the presence of longitudinal measurement invariance.

## Results

### Item analysis

Descriptive statistics showed that the first wave of data used for EFA displayed normal distribution (see Appendix C in the S1 Appendices). Independent *t*-test showed a significant difference between the top 27% and the bottom 27% scoring EFL learners regarding all items (*p* < .05). These results demonstrate the discriminant validity of the items.

### Exploring the factorial structure

**Fig 1:**
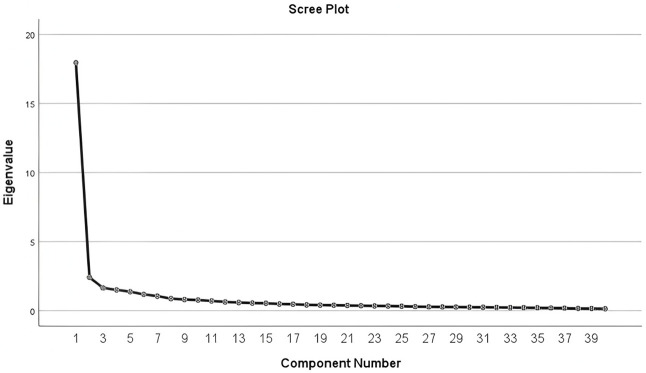
Scree plot of eigenvalues from the exploratory factor analysis of items measuring emotion regulation strategies.

EFA was employed to identify the factorial structure of the questionnaire. The Kaiser-Meyer-Olkin (KMO) measure of sampling adequacy yielded a value of 0.963, and Bartlett’s test of sphericity was found to be significant (χ^2^ = 20339.507, df = 780, *p* < .001), indicating that the dataset was suitable for EFA. A close inspection of the eigenvalues and the scree plot (see Fig 1) suggested a 7-factor solution, which accounted for 67.872% of the total variance of the construct.

Seven items were deleted because they did not load onto the hypothesized latent factors (SM1, SM4, AR1, VRD1, RM1, RM2, and RM3). The remaining thirty-three items were put to EFA, with the number of factors fixed at seven. The results indicate sampling adequacy, with a KMO of 0.957 (χ^2^ = 16003.950, df = 528, *p* < .001). The total variance explained has increased to 70.404%. The initial eigenvalues of the seven extracted factors all exceeded the critical value of 1. All the factor loadings were higher than the benchmark of 0.40 (see Appendix C in S1 Appendices).

The factors were renamed. Factor 1, labeled as *situation selection*, comprises three items reflecting strategies to choose the most desirable situations to avoid negative emotions. Factor 2, labeled as *situation modification*, comprises four items, reflecting strategies to create a desirable learning environment. Factor 3, labeled as *attention redirection*, includes six items reflecting the strategies to allocate attention to pleasant experiences. Factor 4, labeled as *control reappraisal*, with nine items, maintains a sense of control and self-efficacy. Factor 5, labeled as *value upgrading*, includes five items reflecting strategies to emphasize the importance of achievement activities and their outcomes. Factor 6, labeled as *value downgrading*, with three items, downplays the importance of achievement activities and outcomes to seek comfort. Factor 7, labeled as *response modulation*, contains three items that reflect strategies to suppress achievement-related emotions.

### Confirmatory factor analysis

The CFA was conducted with the second wave of data. The first step involved inspecting the items via standardized factor loadings. The CFA results indicate that all items had a factor loading above the threshold value 0.45. Two alternative models were assessed for model fit. One is a first-order structure with seven factors (Model A), and another includes a second-order factor that includes control reappraisal, value upgrading, and value downgrading (Model B). Both models possessed acceptable model fit, with Model A displaying slightly better model fit (see Table 1).

**Table 1:** Comparison of the first-order and second-order models.

	CFI	TLI	RMSEA	SRMR	AIC	ΔAIC
Cut-off value	> 0.90	> 0.90	< 0.08	< 0.08		
Model A	0.924	0.915	0.062	0.050	40959.922	
Model B	0.921	0.914	0.063	0.052	40991.678	31.756

*Note*. Model A is the original first-order seven-factor model. Model B is a second-order model.

The sub-scales were evaluated for internal consistency reliability using Cronbach’s α and composite reliability (CR), while convergent validity was assessed through average variance extracted (AVE), and discriminant validity was determined using the heterotrait-monotrait ratio (HTMT) values. Table 2 presents acceptable levels of reliability (αs > 0.70 and CRs > 0.70), convergent validity (AVE > 0.50), and discriminant validity (HTMT values < 0.85).

**Table 2:** Reliability and validity for emotion regulation strategies.

Dimensions	Reliability		Convergent validity	Latent correlations and discriminant validity						
	α	CR	AVE	1	2	3	4	5	6	7
1. Situation selection	0.796	0.801	0.577		0.642^a^	0.491	0.496	0.505	0.317	0.266
2. Situation modification	0.896	0.895	0.681	0.759^b^		0.658	0.664	0.661	0.431	0.348
3. Attention redirection	0.894	0.897	0.596	0.582	0.734		0.663	0.642	0.550	0.427
4. Control reappraisal	0.937	0.939	0.632	0.574	0.727	0.723		0.728	0.656	0.462
5. Value upgrading	0.913	0.913	0.679	0.592	0.732	0.708	0.787		0.588	0.428
6. Value downgrading	0.829	0.829	0.618	0.387	0.501	0.638	0.743	0.675		0.408
7. Response modulation	0.799	0.802	0.577	0.335	0.410	0.506	0.538	0.505	0.503	

*Note*. a indicates latent correlations between the ER strategies, and b represents the HTMT values.

### Longitudinal measurement invariance over time

Table 3 presents the results of the longitudinal measurement invariance test. According to Chen [53], measurement invariance is supported when ΔCFI ≤ –0.010, ΔRMSEA ≤ 0.015, ΔSRMR ≤ 0.015

**Table 3:** Longitudinal measurement invariance test.

		χ^2^	df	CFI	TLI	RMSEA	SRMR	ΔCFI	ΔRMSEA	ΔSRMR
SS	Configural	44.188	7	0.963	0.920	0.098	0.042			
	Metric	44.970	9	0.964	0.940	0.085	0.044	0.001	−0.013	0.002
	Scalar	65.111	11	0.946	0.926	0.094	0.047	−0.018	0.009	0.003
SM	Configural	66.161	19	0.982	0.973	0.067	0.025			
	Metric	66.341	22	0.983	0.978	0.060	0.025	0.001	−0.007	0.000
	Scalar	70.235	25	0.982	0.980	0.057	0.027	−0.001	−0.003	0.002
AR	Configural	276.667	53	0.940	0.925	0.087	0.036			
	Metric	281.831	58	0.940	0.932	0.084	0.042	0.000	−0.003	0.006
	Scalar	304.292	63	0.935	0.932	0.083	0.044	−0.005	−0.001	0.002
CR	Configural	632.905	131	0.931	0.920	0.083	0.042			
	Metric	635.390	139	0.932	0.925	0.080	0.043	0.001	−0.003	0.001
	Scalar	652.618	147	0.931	0.928	0.079	0.044	−0.001	−0.001	0.001
VRU	Configural	231.741	34	0.950	0.934	0.103	0.044			
	Metric	237.521	38	0.949	0.940	0.098	0.049	−0.001	−0.005	0.005
	Scalar	238.970	42	0.950	0.947	0.092	0.050	0.001	−0.006	0.001
VRD	Configural	36.279	8	0.979	0.960	0.080	0.028			
	Metric	43.848	10	0.975	0.962	0.078	0.049	−0.004	−0.002	0.021
	Scalar	51.380	12	0.970	0.963	0.077	0.042	−0.005	−0.001	−0.007
RM	Configural	56.165	8	0.959	0.924	0.104	0.034			
	Metric	57.750	10	0.960	0.940	0.093	0.039	0.001	−0.011	0.005
	Scalar	59.927	12	0.960	0.950	0.085	0.039	0.000	−0.008	0.000

### Concurrent validity: linkages of ER strategies with L2 emotions

Descriptive statistics indicate that the participants experienced a relatively high level of enjoyment, a moderate to high level of anxiety, and a relatively low level of boredom (see Table 4). The high levels of activating emotions reflect the high value of the test preparation context. In addition, when the CET4 test was approaching, the participants experienced increased anxiety and boredom and decreased enjoyment. The participants generally indicated a moderate to high prevalence of employing ER strategies. Over time, the most used strategies were the strategies of situation selection and situation modification. The use of most er strategies decreased over time, except for value upgrading.

**Table 4:** Descriptive statistics for emotion regulation strategies across time.

	Wave 1				Wave 2			
	*M*	*SD*	Skewness	Kurtosis	*M*	*SD*	Skewness	Kurtosis
Situation Selection	3.96	0.74	−0.20	−0.65	4.00	0.75	−0.21	−0.82
Situation Modification	3.92	0.76	−0.10	−0.64	3.88	0.77	0.06	−0.93
Attention Redirection	3.82	0.75	0.07	−0.67	3.78	0.72	0.20	−0.70
Control Reappraisal	3.89	0.74	−0.02	−0.78	3.72	0.71	0.22	−0.27
Value Upgrading	3.46	0.88	0.04	−0.02	3.81	0.73	0.15	−0.67
Value Downgrading	3.76	0.73	0.07	−0.23	3.48	0.83	0.22	−0.18
Response Modulation	3.52	0.82	0.22	−0.34	3.49	0.79	0.30	−0.30
Anxiety	3.35	0.95	−0.31	−0.06	3.46	0.89	−0.34	0.29
Enjoyment	4.15	0.80	−0.74	0.18	4.10	0.77	−0.53	−0.10
Boredom	2.73	0.90	0.14	−0.03	2.81	0.89	0.18	−0.00

Table 5 shows that all seven ER strategies were significantly and positively correlated with enjoyment at both points in time. Four ER strategies were significantly and negatively correlated with boredom at both points in time. Only one ER strategy (i.e., situation selection) significantly correlated with anxiety at Time 1, but with a positive coefficient. According to L2-specific benchmarks [54], *r* values close to 0.25, 0.40, and 0.60 are small, medium, and large. Most correlations between ER strategies and enjoyment are medium; some are small. Most correlations between ER strategies and boredom are small. All correlations between ER strategies and anxiety are small. Overall, the correlations between ER strategies and enjoyment are larger than those observed in Burić et al. [22], but the correlations between ER strategies and the two negative emotions are smaller than those in Burić et al. [22]. The newly developed L2ERSQ displayed good concurrent criterion validity.

**Table 5:** Latent correlations of ER strategies with L2 emotions.

	Wave 1			Wave 2		
	Anxiety	Enjoyment	Boredom	Anxiety	Enjoyment	Boredom
Situation selection	0.076*	0.399**	−0.088*	0.058	0.047**	−0.091*
Situation modification	−0.045	0.432**	−0.158**	−0.006	0.489**	−0.195**
Attention redirection	0.004	0.362**	−0.052	0.065	0.395**	−0.028
Control re-appraisal	0.008	0.349**	−0.105**	−0.006	0.427**	−0.110**
Value upgrading	0.036	0.444**	−0.224**	0.070	0.483**	−0.163**
Value downgrading	−0.020	0.132**	0.033	0.037	0.193**	0.090*
Response modulation	0.012	0.169**	0.010	0.021	0.117**	0.025

*p < .05; **p < .01.

## Discussion

In response to the validation crisis in L2 research [20], this study aims to develop and validate an L2-specific questionnaire of emotion regulation strategies (L2ERSQ). This study not only provides a reliable instrument for L2 scholars to investigate the use of emotion regulation strategies among L2 learners but also offers insights into the potential of individual strategies for regulating L2 learners’ emotions. Specifically, the results revealed a seven-factor structure of the questionnaire. While this result has largely supported the theoretical propositions of the ERAS model [50], a more refined taxonomy of ER strategies emerged. Specifically, this study further divided cognitive change into three strategies: value upgrading, value downgrading, and control reappraisal. This division refined the strategy of cognitive reappraisal included in existing questionnaires [22,40] and more accurately reflected the regulation of achievement emotions by either upgrading or downgrading the value of an activity or its outcome. Given the critical role of value in shaping language learning and its outcomes, emphasizing or downplaying the value of language learning can have markedly different effects on learners’ emotions. This newly developed questionnaire offers a reliable instrument for future empirical research aimed at understanding the effectiveness of cognitive reappraisal.

This study also expanded existing questionnaires by adopting a positive psychology perspective on emotion regulation. Unlike questionnaires that focus primarily on coping with negative emotions (e.g., CERQ), this study considers proactive strategies to foster positive emotions. For example, the construct of situation selection includes the item, “I change my place of study to study effectively,” while attention redirection includes the item, “When feeling frustrated, I focus my mind on something interesting.” Emphasizing the cultivation of positive emotions is a timely response to recent scholarly interest in the well-being of language learners [55,56].

The L2ERSQ demonstrates reliability and validity at both the indicator and construct levels. The indicators on the scale exhibited factor loadings greater than 0.40. The subscales showed good internal consistency (α > 0.70), composite reliability (CR > 0.70), and convergent validity (AVE > 0.50). The subscales were significantly positively correlated with each other (ranging from 0.317 to 0.728) while maintaining acceptable discriminant validity (HTMT values < 0.85). Additionally, the measurement model exhibited satisfactory overall fit indices (CFI > 0.90, TLI > 0.90, RMSEA < 0.08, and SRMR < 0.08).

The questionnaire has also demonstrated longitudinal measurement invariance, a prerequisite for longitudinal studies examining the reciprocal relationships between emotion regulation strategies and L2 emotions. Advanced statistical methods, such as cross-lagged panel modeling and latent growth modeling, rely on robust, time-invariant measures to rule out the possibility of changes in the instruments over time [57]. The questionnaire can serve as a reliable tool for longitudinal research on the dynamic, reciprocal relationships between ER strategies and L2 emotions. This instrument has significant implications for future research on L2 emotion regulation. It can facilitate longitudinal designs that help establish causal relationships and supports intensive longitudinal studies investigating the dynamics of L2 learners’ efforts in emotion regulation. Such research can complement the evidence generated by existing cross-sectional studies [e.g., 15,42].

This study has provided preliminary evidence on the predictive power of discrete emotion regulation (ER) strategies. Specifically, the subscales exhibited significant positive correlations with L2 enjoyment and generally negative correlations with L2 boredom. These results provide additional empirical support for the potential of emotion regulation strategies in managing learner emotions, particularly highlighting the role of control and value appraisals in shaping these emotions [28,58]. This study demonstrates that value upgrading can enhance enjoyment and reduce boredom, whereas value downgrading can diminish enjoyment and increase boredom. These findings provide deeper insight into the role of value appraisal in regulating L2 emotions. Additionally, the study suggests that attention redirection is positively correlated with enjoyment, consistent with the findings of Man et al. [15]. In other words, redirecting attention to engaging stimuli is an effective method to increase enjoyment during language learning.

However, this study detected only a modest correlation between one emotion regulation (ER) strategy (situation selection) and anxiety. The lack of significant correlations between other ER strategies and anxiety contrasts with findings from previous research. For example, Burić et al. [22] found that seven out of eight ER strategies in their study had significant positive correlations with anxiety. There are two possible explanations for the weak link between emotion regulation and anxiety. First, anxiety has long been shown to have ambivalent effects on language learning [13,14,33]. A moderate level of anxiety is considered essential to avoid failure and achieve desirable outcomes [15,50]. In this sense, eliminating anxiety is not necessarily the goal of emotion regulation among students, especially those aiming for high achievement in high-stakes contexts. Second, anxiety does not always negatively impact emotional well-being, which reflects a balance of positive and negative emotions [48]. In fact, a high level of enjoyment combined with a moderate level of anxiety may indicate emotional well-being in high-stakes contexts [18]. Therefore, the motivation to further reduce anxiety when it is already relatively low is weak. Consequently, the use of emotion regulation strategies is not strongly related to anxiety in the present study.

Despite these insights, this study also has limitations. First, this study relied on self-report data. The respondents might fail to accurately recall or report the strategies they employed. Future research could benefit from incorporating multi-method approaches, such as observational data, qualitative interviews, or physiological measures, to triangulate the findings and assess the effectiveness of emotion regulation strategies. Second, the study only included a cohort of students preparing for a high-stakes English test at a Chinese university. This limitation hinders the generalizability of our findings to other populations. Further research is required to broaden the sampling techniques and participant demographics. It is essential to incorporate students from various backgrounds with different English proficiency levels and learning goals. Third, this study only included seven emotion regulation strategies. While this taxonomy has expanded previous questionnaires, further distinctions can be made in future instrument development. For example, it would be helpful to differentiate between response modulation strategies that aim to release emotions and strategies that aim to suppress the generation or expression of emotions. Alternative emotion regulation strategies such as physical exercise [47] and AI-driven portfolio assessment [39]. Interviews with L2 learners from diverse backgrounds are instrumental in developing a more refined taxonomy of emotion regulation strategies specific to L2 learning. By providing detailed descriptions of the emotion regulation processes, it becomes possible to incorporate L2 learning scenarios into the items representing individual emotion regulation strategies.

## Conclusion

Despite growing interest in emotion regulation within specific learning domains, there are currently no validated emotion regulation questionnaires tailored specifically for L2 research. This article has presented the creation and validation of an L2-specific emotion regulation strategies questionnaire (L2ERSQ). The results have established the factorial structure, reliability, and construct validity of the L2ERSQ. The EFA and CFA results confirmed that the L2ERSQ included seven distinct ER strategies. The significant correlations between ER strategies and L2 enjoyment and boredom across waves of data indicate the concurrent criterion validity of the L2ERSQ. The measurement invariance across time shows the potential of the L2ERSQ in longitudinal research designs, which have been repeatedly called for in emotion regulation research.

This article presents the development and validation of an L2-specific emotion regulation strategies questionnaire (L2ERSQ). The results establish the factorial structure, reliability, and construct validity of the L2ERSQ. Exploratory factor analysis and confirmatory factor analysis confirmed that the L2ERSQ comprises seven distinct emotion regulation strategies. Measurement invariance over time highlights the potential of the L2ERSQ for use in longitudinal research designs, which have been frequently advocated in emotion regulation studies. Significant correlations between these strategies and L2 enjoyment and boredom across multiple data waves demonstrate the concurrent criterion validity of the L2ERSQ.

This article bears practical implications for regulating L2 learners’ emotions. First, the findings can guide language teachers to identify the ER strategies that can affect their students’ emotions. The article indicates that not all ER strategies can bring about desirable affective outcomes. For example, downgrading the value of language learning may increase boredom among students. While individuals may have specific goals for emotion regulation and have varied definitions of desirable emotion [50], some emotions such as boredom are generally recognized as unpleasant and least rewarding. Language teachers and learners would benefit from knowing which ER strategies can make for a desirable emotional state. This study shows that ER strategies were generally not significantly correlated with anxiety. As noted earlier, there are two possible reasons for these insignificant correlations. One is that the listed ER strategies are not effective in regulating anxiety. The other is that L2 learners in challenging learning contexts may not want to indiscriminately eliminate their anxiety. L2 teachers may need to understand their students’ anxiety level and their personal goals of emotion regulation before deciding whether to take proper actions to regulate their students’ anxiety. By contrast, four ER strategies were significantly and negatively correlated with boredom at a small magnitude. That is, ER strategies may have limited power to reduce boredom, though this negative emotion is generally believed to be undesirable. All the ER strategies were significantly and positively correlated with enjoyment, suggesting their potential to enhance the pleasure of language learning.

There are several practical methods to help emotion regulation. For example, L2 teachers may ask students to keep a reflective journal of their emotional experiences and subsequent responses. This specific method can help students become aware of the potential impact of various emotions, the importance of emotion regulation, and the strategies that contribute to their goals of emotion regulation. Another practical method is to organize group discussions, in which students share their emotional experiences and strategies to manage their own emotions. Such sharing sessions, with proper teacher guidance, not only facilitate students’ learning of emotion regulation, but also help L2 teachers to understand the emotional well-being of their students. The sharing sessions undoubtedly function as a channel to release undesirable emotions such as boredom and foster a sense of enjoyment by exchanging interesting personal anecdotes in language learning.

### Consent for publication

All authors approved the final manuscript and the submission to this journal.

## Supporting information

S1 Appendices Questionnaires of Academic Emotion Regulation Strategies and Achievement Emotions. (DOCX)

S2 Dataset Anonymized raw survey data (Excel files) including all variables and labels used in the analyses. (ZIP)
