# Supplementary material for: Developing and validating a measure of L2-specific emotion regulation strategies
Source: PLoS One. 2026 Apr 8;21(4):e0345751. doi: 10.1371/journal.pone.0345751 (PMC13061189; doi:10.1371/journal.pone.0345751)
Supplement: S1 Appendices — (DOCX) [file pone.0345751.s001.docx]

**Appendix A**

Questionnaire of Academic Emotion Regulation Strategies

学习情绪调节策略问卷

1. *挑选学习环境*

*Situation Selection*

SS1. 为了避免干扰，我会选择安静的地方学习英语。

I choose a reliable, non-disruptive place to study English.

SS2. 为了提高英语学习效率，我会主动变更学习地点。

I change my place to study English effectively.

SS3. 我会尽量避免接触高焦虑的同学，以免英语学习受到影响。

I avoid anxious students to avoid feeling anxiety when learning English.

1. *调整学习环境*

*Situation Modification*

SM1. 为了缓解焦虑，我会合理安排学习时间，提高学习效率。

To reduce tension, I will make proper learning plans to study efficiently.

SM2. 为了减少焦虑，我会设定合理的目标，以保证学习进度。

To minimize anxiety, I will set reasonable goals to ensure expected progress.

SM3. 为了缓解学习倦怠，我会预留时间，进行课外活动。

I will spare time for extracurricular activities to alleviate my boredom.

SM4. 为了减少不确定性，我会额外花时间复习英语。

To reduce my uncertainty, I will spend extra time reviewing English.

SM5. 学习内容太多时，我会合理计划以缓解压力。

If the amount of learning material scares me, I carefully organize my schedule of studying.

SM6. 英语考试时，我会调整答题策略，以减轻考试焦虑。

When taking an English test, adjusting the test-taking strategy reduces my test anxiety.

1. *转移注意力*

*Attention Redirection*

AR1. 我会通过回忆成功的经历，减轻对英语考试的焦虑。

I recall successful experiences to reduce my English test anxiety.

AR2. 学习感到沮丧时，我会想一些快乐的事。

When feeling frustrated, I put my mind on something interesting.

AR3. 英语学习无聊时，我会想一些比学习有趣的事。

I start to think about something more fun when studying English becomes boring to me.

AR4. 英语学习无聊时，我会做点有趣的事（例如跟朋友聊天）。

When getting bored with studying English, I do something fun (e.g., talking to a friend).

AR5. 为了减轻学习焦虑，我会放空自己，想想别的事。

When I feel learning anxiety, I ‘shut myself down’ and think of something else.

AR6. 感到英语学习枯燥时，我会将注意力放在自己的兴趣点上。

When I feel bored with English, I will focus on the points that interest me.

AR7. 为了集中注意力，我会设法减少噪音干扰（如戴耳机）。

I try to avoid distraction from noise (e.g., by wearing an earphone).

1. *重新评估学习的价值和意义*

*Value Re-appraisal*

VRU1. 上英语课时，我会提醒自己，即使无聊也要认真听讲。

When I get bored in English class, I remind myself to listen carefully.

VRU2. 走神时，我会提醒自己，英语学习很重要，必须集中注意力。

When my mind is wandering, I remind myself of the importance of learning English and try to concentrate.

VRU3. 感觉英语学习无聊时，我会提醒自己，即使枯燥也要认真学完，争取考试一次通过。

When getting bored with English, I tell myself to be patient with studying to pass the exam at once.

VRU4. 英语学习有困难时，我会告诉自己，要多看几遍。

When confronted with English learning difficulties, I tell myself to study a few more times.

VRU5. 学习懈怠时，我会提醒自己，上课没听懂，课后要解决。

When slacking off, I remind myself to solve the problems encountered in class in time.

VRD1. 上课听不懂时，我会告诉自己，课后总有办法弄懂。

When I don’t understand the lesson, I tell myself that there is always a way to figure it out after the class.

VRD2. 自己看不懂英语时，我会安慰自己，反正上课还会再讲一遍。

When I cannot understand the English materials, I comfort myself with the thought that I’ll go over it again in class anyway.

VRD3. 考试焦虑时，我会安慰自己，考不好也没事，不用紧张。

I reduce exam tension by reminding myself that it is not a big deal to flunk.

VRD4. 考试倦怠时，我会提醒自己，成绩很重要，要认真检查。

When getting tired of the test, I remind myself to check the answer carefully because the score is important.

1. *重新评估控制感*

*Control Re-appraisal*

CR1. 英语学习遇到困难时，我安慰自己别的内容会简单一点，要有信心。

When encountering difficulties in studying English, I tell myself that the rest part will be easier than this.

CR2. 英语学习遇到困难时，我会安慰自己，多学几遍就会了。

When encountering difficulties in English learning, I comfort myself that practice makes perfect.

CR3. 学习遇到困难时，我会安慰自己，英语考试不会这么难，要对自己有信心。

When having learning difficulties, I tell myself that the English exam will not be so hard.

CR4. 我会通过心理暗示，提升自己完成任务的信心。

I boost my confidence in accomplishing tasks through positive self-talk.

CR5. 焦虑时，我会列出自己取得的进展，提升学习成就感。

I alleviate my anxiety by listing the progress I have made to boost a sense of achievement.

CR6. 英语学习进展不顺利时，我会分析客观原因，例如宿舍太吵，影响我学习。

When my English studies are not going well, I attribute it to external factors such as the dormitory being too noisy, which affects my studies.

CR7. 英语考试前，我会提醒自己复习已到位，要对考试有信心。

When I am about to sit the English exam, I remind myself that I have sufficiently prepared for it to boost my self-confidence.

CR8. 英语考试时，我会给自己打气，相信自己可以解决难题。

When sitting the English exam, I tell myself that I can make it.

CR9. 英语成绩不理想时，我会分析客观原因，比如题目太难。

When getting unsatisfactory scores in English, I attribute it to objective reasons, such as the questions being too complicated.

1. *调节情绪反应*

*Response Modulation*

RM1. 焦虑时，我会努力保持冷静，微笑面对。

When getting anxious, I try my best to stay calm and smile.

RM2. 我会通过深呼吸、冥想等方式来缓解焦虑。

I relieve anxiety by breathing deeply or meditating.

RM3. 我会通过运动、哭泣、吃东西等方式来宣泄焦虑。

I release my anxiety by exercising, crying, and eating.

RM4. 我会控制自己的情绪，喜怒不形于色。

I control my emotions and do not show them to others.

RM5. 有什么情绪，我都会藏在心里。

I keep my emotions to myself.

RM6. 有消极情绪时，我会努力不在人前流露。

When I have negative emotions, I try not to show them to people.

**Appendix B**

Questionnaire of Achievement Emotions

二语情绪问卷

1. 备考英语时，我会焦虑。

I get anxious while studying for an English exam.

1. 备考英语时，我会担心没能完全理解所复习的内容。

After studying for an English exam, I worry whether I have properly understood the materials.

1. 我内心会忐忑不安，因为我感觉没复习好。

I worry about the exam because I’m not fully prepared.

1. 英语考试前，我担心自己没准备好，会考砸。

Before taking an English exam, I worry that I might fail because of insufficient preparation.

1. 我一坐下来备考英语，就感到无聊和疲倦。

While studying for an English exam, I get bored and tired sitting at my desk.

1. 英语学习很无聊，我希望把功课推迟到第二天去做。

Because studying English is boring, I would rather put off this boring work till tomorrow.

1. 备考英语时，我发现自己会走神。

While studying for an English test, I find my mind wandering.

1. 备考英语时我会觉得无聊，感觉时间过得很慢。

While studying for an English test, I spend my time thinking of how time stands still on this boring material.

1. 英语学习顺利时，我感到心情愉悦。

While studying for an English exam, I get excited when my studies are going well.

1. 备考英语时，学到新知识让我开心。

While studying for an English exam, I enjoy acquiring new knowledge.

1. 备考英语时，感到自己英语有进步让我开心。

After studying for an English exam, I am so happy about the progress I made.

1. 英语学习取得进步让我开心，我有动力继续学习。

I am so happy about the progress in English I made that I am motivated to continue studying.

**Appendix C**

Table 1 Results of normality test of all items

|  | Minimum | Maximum | Mean | *SD* | Skewness | | Kurtosis | |
| --- | --- | --- | --- | --- | --- | --- | --- | --- |
|  | Statistic | Statistic | Statistic | Statistic | Statistic | SE | Statistic | SE |
| SS1 | 1.0 | 5.0 | 4.121 | .8794 | -.743 | .124 | .086 | .247 |
| SS2 | 1.0 | 5.0 | 3.851 | .9874 | -.457 | .124 | -.357 | .247 |
| SS3 | 1.0 | 5.0 | 3.456 | 1.0421 | -.145 | .124 | -.348 | .247 |
| SM1 | 1.0 | 5.0 | 3.773 | .9291 | -.214 | .124 | -.706 | .247 |
| SM2 | 1.0 | 5.0 | 3.763 | .8924 | -.152 | .124 | -.518 | .247 |
| SM3 | 1.0 | 5.0 | 3.706 | .9544 | -.028 | .124 | -.906 | .247 |
| SM4 | 1.0 | 5.0 | 3.655 | .9118 | -.023 | .124 | -.597 | .247 |
| SM5 | 1.0 | 5.0 | 3.709 | .8980 | -.125 | .124 | -.413 | .247 |
| SM6 | 1.0 | 5.0 | 3.765 | .9060 | -.169 | .124 | -.470 | .247 |
| AR1 | 1.0 | 5.0 | 3.497 | .9712 | -.061 | .124 | -.339 | .247 |
| AR2 | 1.0 | 5.0 | 3.668 | .9180 | -.139 | .124 | -.285 | .247 |
| AR3 | 1.0 | 5.0 | 3.642 | .9082 | -.063 | .124 | -.451 | .247 |
| AR4 | 1.0 | 5.0 | 3.786 | .8615 | -.158 | .124 | -.394 | .247 |
| AR5 | 1.0 | 5.0 | 3.763 | .8448 | -.148 | .124 | -.287 | .247 |
| AR6 | 1.0 | 5.0 | 3.714 | .8341 | -.120 | .124 | -.077 | .247 |
| AR7 | 1.0 | 5.0 | 3.644 | .9895 | -.220 | .124 | -.447 | .247 |
| VRU1 | 1.0 | 5.0 | 3.799 | .8450 | -.018 | .124 | -.774 | .247 |
| VRU2 | 1.0 | 5.0 | 3.791 | .8446 | .049 | .124 | -.849 | .247 |
| VRU3 | 1.0 | 5.0 | 3.889 | .8808 | -.215 | .124 | -.592 | .247 |
| VRU4 | 1.0 | 5.0 | 3.747 | .8910 | -.077 | .124 | -.577 | .247 |
| VRU5 | 1.0 | 5.0 | 3.794 | .8498 | -.100 | .124 | -.546 | .247 |
| VRD1 | 1.0 | 5.0 | 3.691 | .9020 | -.157 | .124 | -.293 | .247 |
| VRD2 | 1.0 | 5.0 | 3.271 | 1.0350 | -.125 | .124 | -.297 | .247 |
| VRD3 | 1.0 | 5.0 | 3.479 | .9357 | -.178 | .124 | -.066 | .247 |
| VRD4 | 1.0 | 5.0 | 3.549 | .8864 | .052 | .124 | -.332 | .247 |
| CR1 | 1.0 | 5.0 | 3.696 | .8688 | .037 | .124 | -.729 | .247 |
| CR2 | 1.0 | 5.0 | 3.763 | .8325 | .009 | .124 | -.684 | .247 |
| CR3 | 1.0 | 5.0 | 3.673 | .8588 | .069 | .124 | -.683 | .247 |
| CR4 | 2.0 | 5.0 | 3.755 | .8380 | .063 | .124 | -.884 | .247 |
| CR5 | 1.0 | 5.0 | 3.665 | .8899 | -.042 | .124 | -.498 | .247 |
| CR6 | 1.0 | 5.0 | 3.423 | .9892 | -.235 | .124 | -.070 | .247 |
| CR7 | 1.0 | 5.0 | 3.706 | .8724 | -.026 | .124 | -.482 | .247 |
| CR8 | 1.0 | 5.0 | 3.784 | .8350 | .077 | .124 | -.831 | .247 |
| CR9 | 1.0 | 5.0 | 3.526 | .9438 | -.186 | .124 | -.083 | .247 |
| RM1 | 1.0 | 5.0 | 3.727 | .8491 | -.006 | .124 | -.543 | .247 |
| RM2 | 1.0 | 5.0 | 3.660 | .9222 | -.029 | .124 | -.555 | .247 |
| RM3 | 1.0 | 5.0 | 3.536 | 1.0596 | -.429 | .124 | -.162 | .247 |
| RM4 | 1.0 | 5.0 | 3.482 | .9710 | -.153 | .124 | -.208 | .247 |
| RM5 | 1.0 | 5.0 | 3.358 | .9794 | .012 | .124 | -.243 | .247 |
| RM6 | 1.0 | 5.0 | 3.582 | .9041 | -.174 | .124 | -.075 | .247 |

Table 2 Results of EFA (Pattern matrix)

|  | Factors | | | | | | |
| --- | --- | --- | --- | --- | --- | --- | --- |
|  | 1 | 2 | 3 | 4 | 5 | 6 | 7 |
| CR3 | .739 |  |  |  |  |  |  |
| CR4 | .688 |  |  |  |  |  |  |
| CR5 | .688 |  |  |  |  |  |  |
| CR7 | .681 |  |  |  |  |  |  |
| CR2 | .678 |  |  |  |  |  |  |
| CR1 | .649 |  |  |  |  |  |  |
| CR9 | .633 |  |  |  |  |  |  |
| CR6 | .614 |  |  |  |  |  |  |
| CR8 | .611 |  |  |  |  |  |  |
| AR4 |  | .782 |  |  |  |  |  |
| AR6 |  | .762 |  |  |  |  |  |
| AR5 |  | .718 |  |  |  |  |  |
| AR3 |  | .710 |  |  |  |  |  |
| AR7 |  | .590 |  |  |  |  |  |
| AR2 |  | .530 |  |  |  |  |  |
| VU1 |  |  | .755 |  |  |  |  |
| VU2 |  |  | .753 |  |  |  |  |
| VU3 |  |  | .744 |  |  |  |  |
| VU5 |  |  | .662 |  |  |  |  |
| VU4 |  |  | .491 |  |  |  |  |
| SM3 |  |  |  | .746 |  |  |  |
| SM5 |  |  |  | .716 |  |  |  |
| SM6 |  |  |  | .642 |  |  |  |
| SM2 |  |  |  | .639 |  |  |  |
| VD3 |  |  |  |  | .792 |  |  |
| VD2 |  |  |  |  | .750 |  |  |
| VD4 |  |  |  |  | .719 |  |  |
| RM5 |  |  |  |  |  | .847 |  |
| RM6 |  |  |  |  |  | .771 |  |
| RM4 |  |  |  |  |  | .689 |  |
| SS1 |  |  |  |  |  |  | .781 |
| SS2 |  |  |  |  |  |  | .705 |
| SS3 |  |  |  |  |  |  | .681 |
| Extraction Method: Principal Component Analysis.  Rotation Method: Varimax with Kaiser Normalization. | | | | | | | |
| a. Rotation converged in 7 iterations. | | | | | | | |
